# Synthesis of hexagonal structured wurtzite and chalcopyrite CuInS_2 _via a simple solution route

**DOI:** 10.1186/1556-276X-6-562

**Published:** 2011-10-25

**Authors:** Xia Sheng, Lei Wang, Yeping Luo, Deren Yang

**Affiliations:** 1 State Key Laboratory of Silicon Materials and Department of Materials Science and Engineering, Zhejiang University, Hangzhou 310027, People's Republic of China

**Keywords:** wurtzite, chalcopyrite, hexagonal structure, CuInS_2_

## Abstract

Wurtzite semiconductor CuInS_2 _[CIS] has been reported in recent years. As a kind of metastable structure, it is a great challenge to synthesize pure wurtzite CIS at low temperature. In this paper, via a simple and quick solution route, we synthesize both wurtzite- and chalcopyrite-structure CIS. Well-controlled wurtzite CIS hexagonal plates are obtained when an appropriate agent is added. The influence of the used agent triethanolamine [TEA] has also been studied, and it turns out that without TEA, chalcopyrite CIS with a kind of rare morphology is formed through this method.

## Introduction

Ternary • I-III-VI_2 _semiconductors have garnered great interest due to their promising photovoltaic applications [1,2]. Meanwhile, the growing need for highly efficient and low-cost photovoltaic devices continues to drive new research in developing non-vacuum techniques. Thus, solution routes to fabricate I-III-VI_2 _semiconductor nanocrystals have been greatly developed because nanocrystal synthesis can utilize lower-cost processing and device fabrication can benefit from roll-to-roll or solution-phase processing such as spin-coating [3-10]. Among the kinds of I-III-VI_2 _semiconductors, one good example is CuInS_2 _[CIS] which has high optical absorption coefficient (>10^5 ^cm^-1^) and desirable bandgap (approximately 1.45 eV) that matches well with solar spectra [11,12]. Therefore, researches on nanocrystal CIS synthesis have attached great attention [13-18].

It was reported that CIS has three crystal structures [19,20]: these are (1) the chalcopyrite structure [CH-CIS], stable from room temperature to 1,253 K; (2) the zinc-blende structure, stable between 1,253 and 1,318 K; and (3) an unknown structure, existing from 1,318 K to melting temperature. It was found that the unknown structure could be turned into wurtzite phase, while Cu and In atoms occupy the cation sublattice positions disorderly [21]. Moreover, previous studies have proved that both the zinc-blende and wurtzite structures are metastable at room temperature since they may transform into chalcopyrite phase as the temperature recurred to room temperature [19,20]. As a result, CH-CIS is believed to be the most common phase and extensively used in CIS solar cells.

Recently, Pan et al. reported the synthesis of zinc-blende- and wurtzite-structure CIS [WZ-CIS] nanocrystals by a hot-injection method [22], which brings great interest in these two structures, especially in WZ-CIS. Since wurtzite phase allows flexibility of stoichiometry, it provides the ability to tune the Fermi energy over a wide range, which is beneficial for device fabrication [23]. Some groups have reported to synthesize WZ-CIS nanocrystals in recent years [23-29]. Most reported works are also synthesized in an oil system since former researches find out that solvents like ethanolamine, ethylenediamine, and isopropanolamine are beneficial for the formation of WZ-CIS [21]. However, it is still a challenge for the synthesis of metastable WZ-CIS at room temperature, especially via a simple method and low-cost precursors.

In our study, we synthesize pure and well-controlled WZ-CIS via a simple and quick solution route in a polyalcohol system under low temperature. We find that the agent triethanolamine [TEA] plays an important role in the synthesis of WZ-CIS phase. Without TEA, large-diameter hexagonal-structure CH-CIS is obtained. It is a kind of rare morphology in CH-CIS since most other groups pay more attention to controlling the sizes and shapes of the particles and focus on the synthesis of nanosheets, nanorods, quantum dots, and others [13-18]. The growth process and mechanism of hexagonal-structure CH-CIS are discussed.

## Experimental details

### Chemicals

All the reagents are used as received without any further purification. The reagents are as follows: copper(I) dichloride [CuCl 2H_2_O], indium(III) trichloride [InCl_3 _4H_2_O], thiourea [TA], diethylene glycol [DEG], and TEA.

### Preparation

Nanostructured WZ-CIS samples are synthesized via a simple and quick solution route. One mmol of CuCl 2H_2_O and 1 mmol of InCl_3 _4H_2_O are dissolved into 40 mL DEG in a three-neck flask. This solution is stirred under N_2_, while the temperature rises to 180°C. After adding 3 mL TEA while stirring, the solution turns into a deeper color but still clear without any precipitation. Then, stoichiometric amounts of TA dissolved in 10 mL DEG is slowly added into the former solution. After a 2-h reaction at 180°C, the flask is removed from the heater and cooled at room temperature. The precipitates are separated by centrifugation, washed with ethanol for three to five times, and dried at 80°C for 5 h. Meanwhile, CH-CIS samples are synthesized via a similar route, but without adding TEA.

### Characterization

The as-prepared products are characterized by X-ray diffraction [XRD], scanning electron microscopy [SEM], and transmission electron microscopy [TEM]. XRD is carried out to study the crystal structures of all the samples by using an X'Pert PRO (PANalytical, Almelo, The Netherlands) diffractometer equipped with a Cu Kα radiation source. Data are collected by step-scanning of 2θ from 10° to 70° with a step of 0.02° and a counting time of 1 s per step. Morphology of the products is investigated by SEM and TEM. The SEM images are taken by SEM S4800 (Hitachi, Tokyo, Japan). The TEM images and high resolution TEM [HRTEM] are acquired by Tecnai F20 (FEI, Hillsboro, OR, USA).

## Results and discussion

### Structure characterization of WZ-CIS

**Figure 1:**
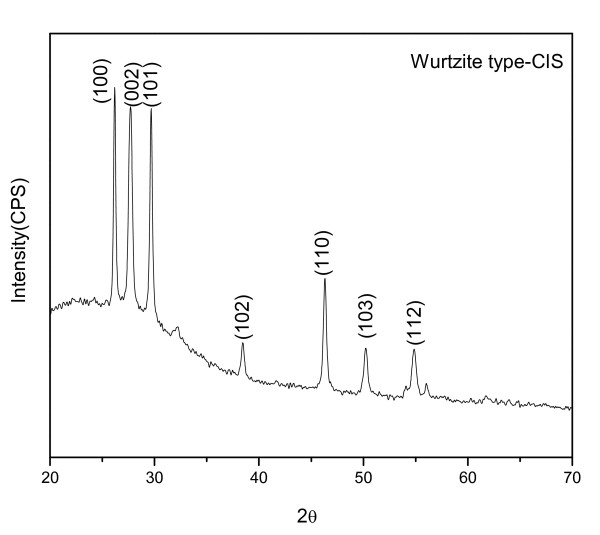
**XRD pattern of the WZ-CIS particles with TEA**.

The products are studied by XRD to refine the structure. A calculated pattern using the lattice parameters reported by Pan et al. (unit cell dimensions a = b = 3.897 Å, c = 6.441 Å and space group: P63mc) [22] has a good match to the experimental XRD pattern (Figure 1), which indicates that the products are wurtzite-structure CIS. All the diffraction peaks have a good match to the previous reported wurtzite CIS pattern [22], and no chalcopyrite CIS phase is determined.

**Figure 2:**
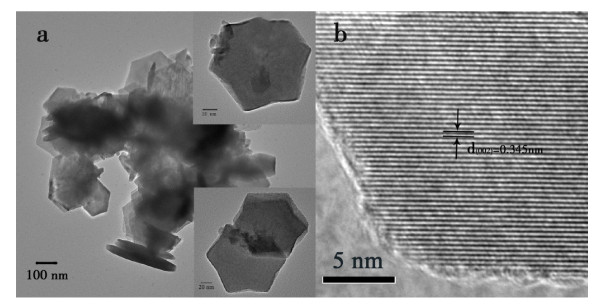
**WZ-CIS particles**. (**a**) TEM and (**b**) HRTEM images of the WZ-CIS particles.

The morphologies of the samples are examined by TEM and HRTEM. Figure 2 shows the typical images of the products, which indicate that the products are hexagonal nanostructured plates. The average diameter of the plates is 80 to 100 nm, and the thickness is about 30 nm. This kind of nanostructures has also been reported by other groups using solvothermal methods or other oil system solvents [21,24]. The HRTEM image (Figure 2b) exhibits clear lattice fringes with a spacing of 0.345 nm, which is well matched to the interplanar spacing of (002) plane of the WZ-CIS.

### The influence of TEA

A former study has reported that solvents like ethanolamine, ethylenediamine, and isopropanolamine are beneficial for the formation of WZ-CIS [24,25]. The pivot is that these solvents play important roles as ligand and reducing agent which reduce Cu^2+ ^to Cu^+^. Qi et al. have synthesized WZ-CIS in 2009 using ethanolamine as solvent [21]. They claim that the successful synthesis of WZ-CIS strongly depends on the formation of coordination between Cu^2+ ^or Cu^+ ^and -NH_2_. In our work, TEA is the pivotal agent. TEA is a kind of versatile ligand that readily forms coordination compounds with almost all metal ions [30]. It can play a similar role as ethanolamine, which is coordinating with Cu^+ ^and facilitating the formation of WZ-CIS. Also, the experiment phenomena show clearly that only when the Cu source and TEA have an appropriate ratio and form a clear solution before the S source is added can pure WZ-CIS be obtained. Otherwise, when the precursor solution is turbid, CH-CIS will co-exit with WZ-CIS. Moreover, TEA also provides ligand for In^3+ ^to limit the size and control the morphology of the products. It can be presumed that TEA coordinates with Cu^+ ^and In^3+^, changing and controlling the relationship of release rates between these two cations, and makes Cu^+ ^and In^3+ ^occupy the cation sublattice positions disorderly when they react with S^2-^.

**Figure 3:**
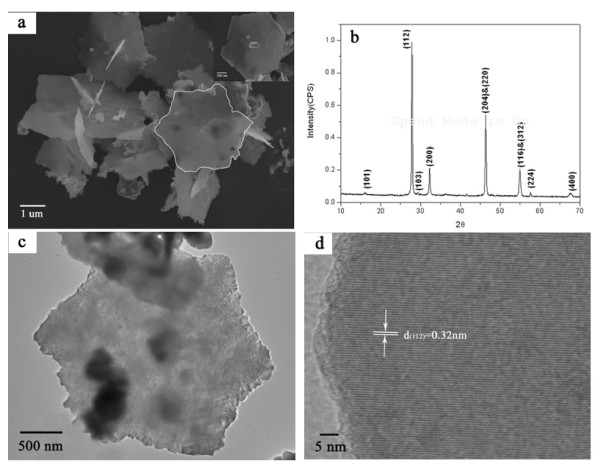
**CH-CIS particles without TEA**. (**a**) SEM, (**b**) XRD pattern, (**c**) TEM, and (**d**) HRTEM of the CH-CIS particles without TEA.

Figure 3 shows the XRD and SEM results of the products using the same synthesis method, but not adding TEA. In comparison with those with TEA (as shown in Figures 1 and 2), two significant differences can be found. First, without using TEA, the products appear as chalcopyrite phase. As seen from the XRD pattern (Figure 3b), all the diffraction peaks are perfectly matched with tetragonal lattice CIS (JCPDS PCPDFWIN, No. 85-1575). Second, the hexagonal plate morphology has remained, but the plates are much bigger in size and have a diameter of 2 to 3 μm and a thickness of about 100 nm (Figure 3a). The HRTEM image (Figure 3d) exhibits (112) orientation of CH-CIS with a d-spacing of 0.32 nm. These two differences indicate that the -NH_2 _in TEA molecules plays a key role to synthesize WZ-CIS and control the size of the CIS hexagonal plates.

### The growth process of CH-CIS hexagonal plates

**Figure 4:**
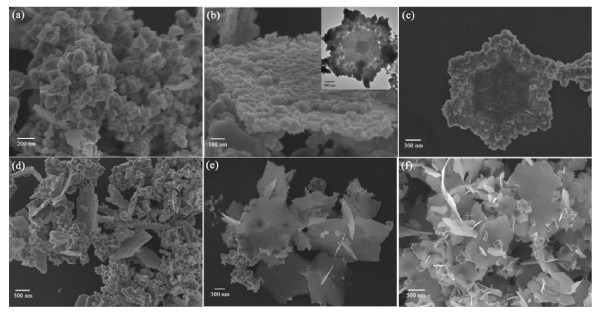
**Growth process of CH-CIS particles**. SEM images of products reacting at (**a**) 5, (**b**, **c**) 10, (**d**) 15, (**e**) 30, and (**f**) 60 min; insert: TEM images at 10 min.

As CH-CIS belongs to a tetragonal lattice, to investigate the growth process of the hexagonal plates, the samples obtained after different reaction times are characterized by SEM and TEM (Figure 4a, b, c, d, e, f). It can be seen that the hexagonal structure is fabricated by self-aggregated nanoparticles about 100 nm in size. Both the SEM and TEM images (the insert of Figure 4) verify this self-aggregation process. As the reaction is going on, the aggregated nanoparticles recrystallize from the center of the plates, forming a smooth surface (the dark central part of Figure 4c). After 30-min reaction, most of the products are hexagonal plates (Figure 4e).

**Figure 5:**
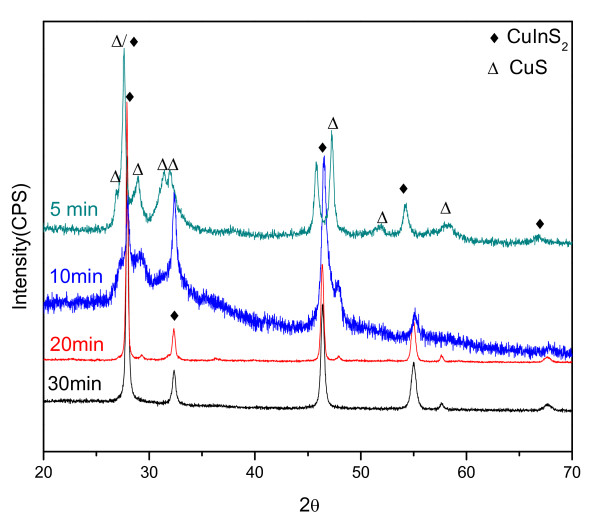
XRD patterns of CH-CIS particles reacting at different times

The XRD patterns of the products at different reaction times shown in Figure 5 explain the growth process further. After 5-min reaction, most of the diffractions that peak in the XRD pattern are indexed to hexagonal phase CuS with CH-CIS phase. As the time is expanding, the diffraction peaks of CH-CIS get stronger while the peaks of CuS become weaker. After 20 min, nearly pure phase CH-CIS is formed with little CuS existence. This kind of reaction process has also been reported by other groups in the synthesis of flower-like CIS particles [31]. It indicates that hexagonal CuS is formed first. CuS nanoparticles grow into small flakes, which exhibit the growth characteristic habit for hexagonal CuS. Then, CuS flakes self-aggregate to form hexagonal plates while at the same time, they react with In^3+ ^to form CIS. As the time is expanding, CuS decreases while CIS increases. At the end, the CH-CIS pure phase is formed.

## Conclusion

In conclusion, WZ-CIS with a well-controlled hexagonal structure is synthesized via a simple and quick solution route. It is found that the agent TEA plays a key role in forming the wurtzite phase and controlling the size of the products since it coordinates with Cu^+ ^and also provides ligand for In^3+ ^to limit and control the morphology of the product. Without TEA, the products appeared as CH-CIS phase, and the hexagonal structures had much larger diameters. The growth process shows that the fabrication of CH-CIS hexagonal structures is due to hexagonal phase CuS self-aggregation and reaction with In^3+^. After a short time, about 20 min, nearly pure CH-CIS phase with hexagonal structures is formed.

## Competing interests

The authors declare that they have no competing interests.

## Authors' contributions

All the authors contributed to the writing of the manuscript. XS and YL carried out the experiments under the instruction of LW and DY. Also, the financial support was provided by DY. All the authors read and approved the final manuscript.

## References

[B1] Habas SE Platt HAS van Hest MFAM Ginley DS Low-cost inorganic solar cells: from ink to printed device Chem Rev 2010 110 6571 10.1021/cr100191d 20973478

[B2] Editors Call for papers: special issue on chalcopyrite thin film solar cells Prog Photovolt: Res Appl 2008 16 271

[B3] Panthani MG Akhavan V Goodfellow B Schmidtke JP Dunn L Dodabalapur A Barbara PF Korgel BA Synthesis of CuInS_2_, CuInSe_2_, and Cu(In_x_Ga_1-x_)Se_2 _(CIGS) nanocrystal "inks" for printable photovoltaics J Am Chem Soc 2008 130 16770 10.1021/ja805845q 19049468

[B4] Norsworthy G Leidholm CR Halani A Kapur VK Roe R Basol BM Matson R CIS film growth by metallic ink coating and selenization Solar Energy Materials & Solar Cells 2000 60 127 10.1016/S0927-0248(99)00075-622030726

[B5] Hibberd CJ Chassaing E Liu W Mitzi DB Lincot D Tiwari N Non-vacuum methods for formation of Cu(In, Ga)(Se, S)_2 _thin film photovoltaic absorbers Prog Photovolt: Res Appl 2010 18 434 10.1002/pip.914

[B6] Long F Wang W Tao H Jia T Li X Zou Z Fu Z Solvothermal synthesis, nanocrystal print and photoelectrochemical properties of CuInS_2 _thin film Materials Letters 2010 64 195 10.1016/j.matlet.2009.10.044

[B7] Weil BD Connor ST Cui Y CuInS_2 _solar cells by air-stable ink rolling J Am Chem Soc 2010 132 6642 10.1021/ja1020475 20423082

[B8] Guo Q Kim SJ Kar M Birkmire WNSRW Stach EA Agrawal R Hillhouse HW Development of CuInSe_2 _nanocrystal and nanoring inks for low-cost solar cells Nano Letters 2008 8 2982 10.1021/nl802042g 18672940

[B9] Chen G Wang L Sheng X Yang D Cu-In intermetallic compound inks for CuInS_2 _solar cells J Mater Sci: Mater Electron

[B10] Redinger D Molesa S Shong Y Farschi R Subramanian V An ink-jet-deposited passive component process for RFID IEEE Trans Electron Devices 2004 51 1978 10.1109/TED.2004.838451

[B11] Bandyopadhyaya S Chaudhuri S Pal AK Synthesis of CuInS_2 _flms by sulphurization of Cu/In stacked elemental layers Solar Energy Materials & Solar Cells 2000 60 323 10.1016/S0927-0248(99)00064-122030726

[B12] Klaer J Bruns J Henninger R Siemer K Klenk R Ellmer K Bräunig D Efficient CuInS_2 _thin-film solar cells prepared by a sequential process Semicond Sci Technol 1998 13 1456 10.1088/0268-1242/13/12/022

[B13] Zhong H Zhou Y Ye M He Y Ye J He C Yang C Li Y Controlled synthesis and optical properties of colloidal ternary chalcogenide CuInS_2 _nanocrystals Chem Mater 2008 20 6434 10.1021/cm8006827

[B14] Chang C Ting J Phase, morphology, and dimension control of CIS powders prepared using a solvothermal process Thin Solid Films 2009 517 4174 10.1016/j.tsf.2009.02.037

[B15] Kruszynska M Borchert H Parisi J Kolny-Olesiak Synthesis and shape control of CuInS_2 _nanoparticles J Am Chem Soc 2010 132 15976 10.1021/ja103828f 20958030

[B16] Castro SL Bailey SG Raffaelle RP Banger KK Hepp AF Synthesis and characterization of colloidal CuInS_2 _nanoparticles from a molecular single-source precursor J Phys Chem B 2004 108 12429 10.1021/jp049107p

[B17] Courtel FM Hammami A Imbeault R Hersant G Paynter RW Marsan B Morin M Synthesis of n-type CuInS_2 _particles using N-methylimidazole, characterization and growth mechanism Chem Mater 2010 22 3752 10.1021/cm100750z

[B18] Han S Kong M Guo Y Wang M Synthesis of copper indium sulfide nanoparticles by solvothermal method Materials Letters 2009 63 1192 10.1016/j.matlet.2009.02.032

[B19] Binsma JJM Giling LJ Bloem J Phase relations in the system Cu_2_S-In_2_S_3_ J Cryst Growth 1980 50 429 10.1016/0022-0248(80)90090-1

[B20] Abrahams SC Berbstein JL Piezoeletric nonlinear optic CuGaS_2 _and CuInS_2 _crystal structure: sublattice distortion in AIBIIICVI_2 _and AIIBIVCV_2 _type chalcopyrites The Journal of Chemical Physics 1973 59 5415 10.1063/1.1679891

[B21] Qi Y Liu Q Tang K Liang Z Ren Z Liu X Synthesis and characterization of nanostructured wurtzite CuInS_2_: a new cation disordered polymorph of CuInS_2_ J Phy Chem C 2009 113 3939 10.1021/jp807987t

[B22] Pan D An L Sun Z Hou W Yang Y Yang Z Lu Y Synthesis of Cu-In-S ternary nanocrystals with tunable structure and composition J Am Chem Soc 2008 130 5620 10.1021/ja711027j 18396869

[B23] Connor ST Hsu C Weil BD Aloni BD Cui Y Phase transformation of biphasic Cu_2_S-CuInS_2 _to monophasic CuInS_2 _nanorods J Am Chem Soc 2009 131 4962 10.1021/ja809901u 19281233

[B24] Koo B Patel RN Korgel BA Wurtzite-chalcopyrite polytypism in CuInS_2 _nanodisks Chem Mater 2009 21 1962 10.1021/cm900363w

[B25] Norako ME Franzman MA Brutchey RL Growth kinetics of monodisperse Cu-In-S nanocrystals using a dialkyl disulfide sulfur source Chem Mater 2009 21 4299 10.1021/cm9015673

[B26] Lu X Zhuang Z Peng Q Li Y Controlled synthesis of wurtzite CuInS_2 _nanocrystals and their side-by-side nanorod assemblies Cryst Eng Comm

[B27] Bera P Seok S Facile synthesis of nanocrystalline wurtzite Cu-In-S by amine-assisted decomposition of precursors Journal of Solid State Chemistry 2010 183 3669

[B28] Kruszynska M Borchert H Parisi J Kolny-Olesiak J Synthesis and shape control of CuInS_2 _nanoparticles J Am Chem Soc 2010 132 15976 10.1021/ja103828f 20958030

[B29] Batabyal SK Tian L Venkatram N Ji W Vittal JJ Phase-selective synthesis of CuInS_2 _nanocrystals J Phys Chem C 2009 113 15037 10.1021/jp905234y

[B30] Karadag A Yilmaz VT Thoene C Di- and triethanolamine complexes of Co(II), Ni(II), Cu(II) and Zn(II) with thiocyanate: synthesis, spectral and thermal studies. Crystal structure of dimeric Cu(II) complex with deprotonated diethanolamine, [Cu_2_(μ-dea)_2_(NCS)_2_] Polyhedron 2001 20 635 10.1016/S0277-5387(01)00720-3

[B31] Zheng L Xu Y Song Y Wu C Zhang M Xie Y Monodisperse CuInS_2 _hierarchical microarchitectures for photocatalytic H_2 _evolution under visible light Inorg Chem 2009 48 4003 10.1021/ic802399f 19341303

